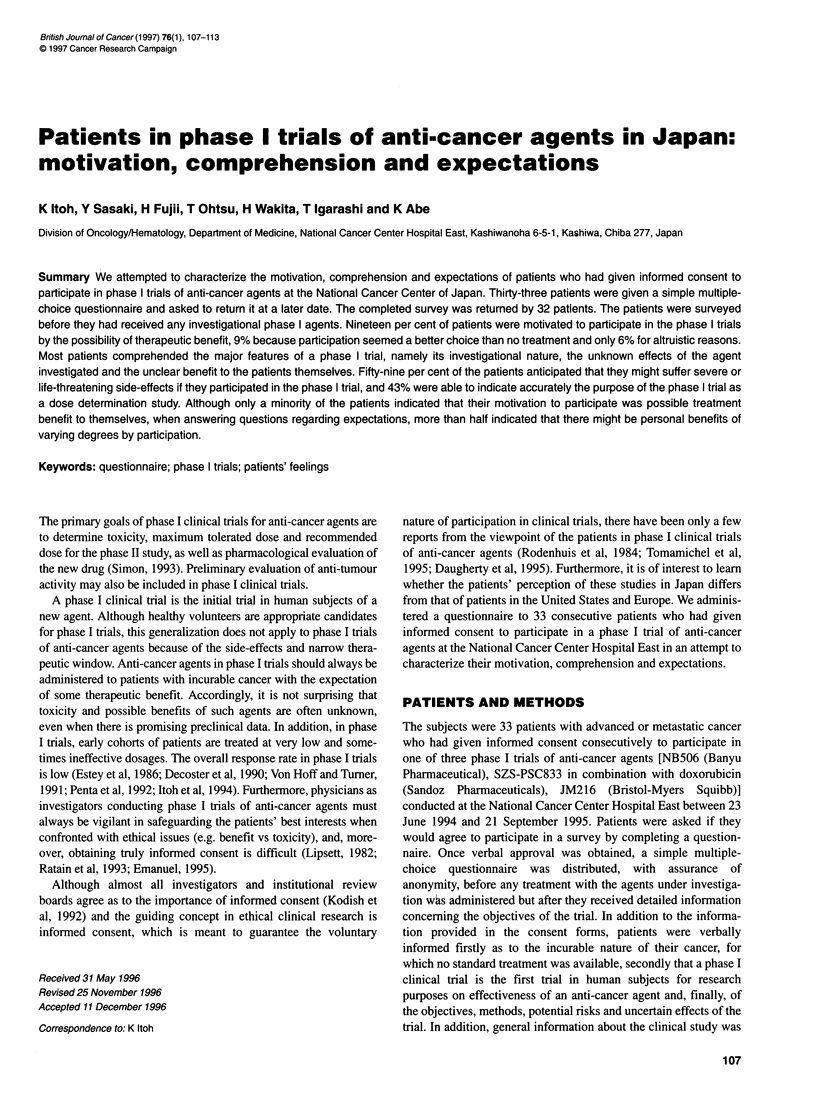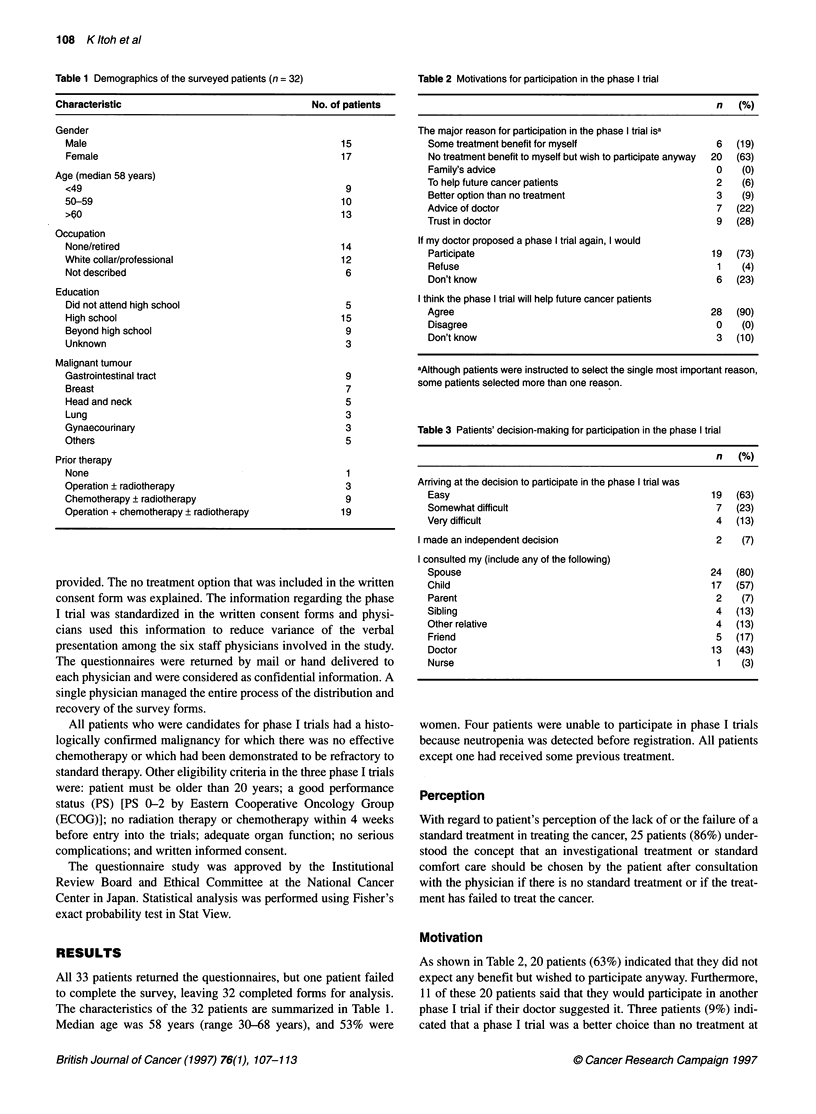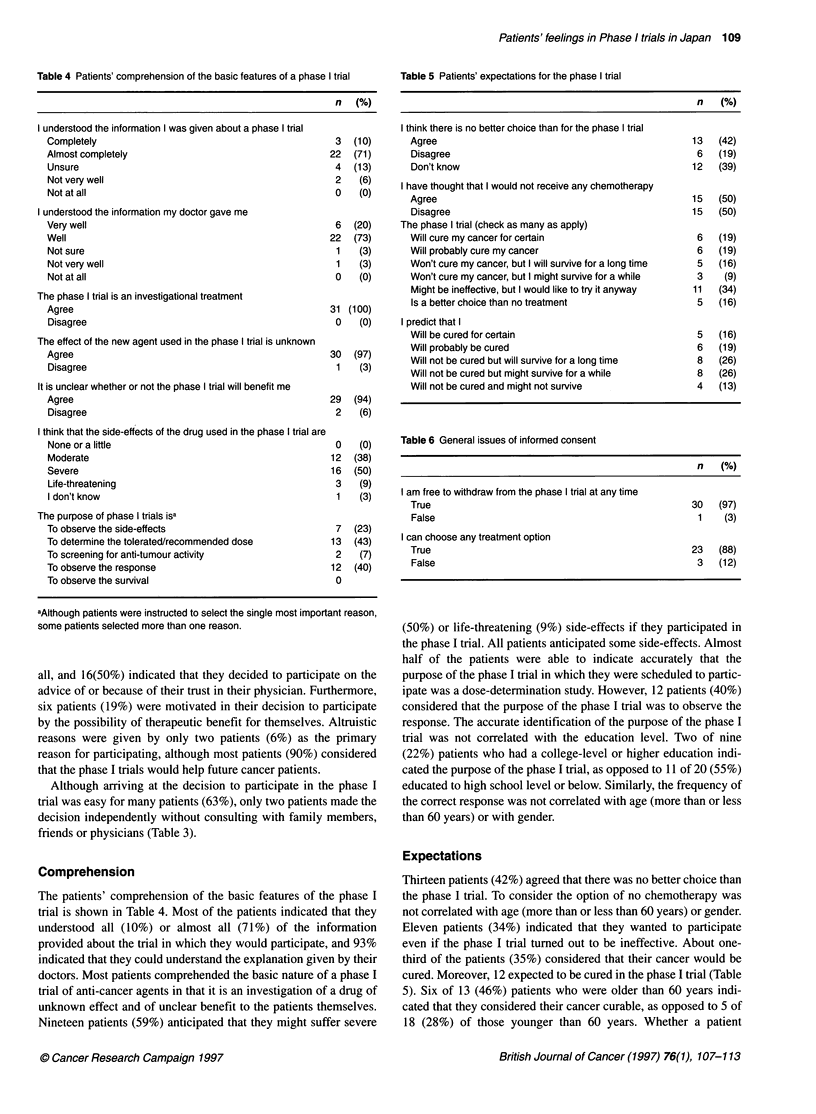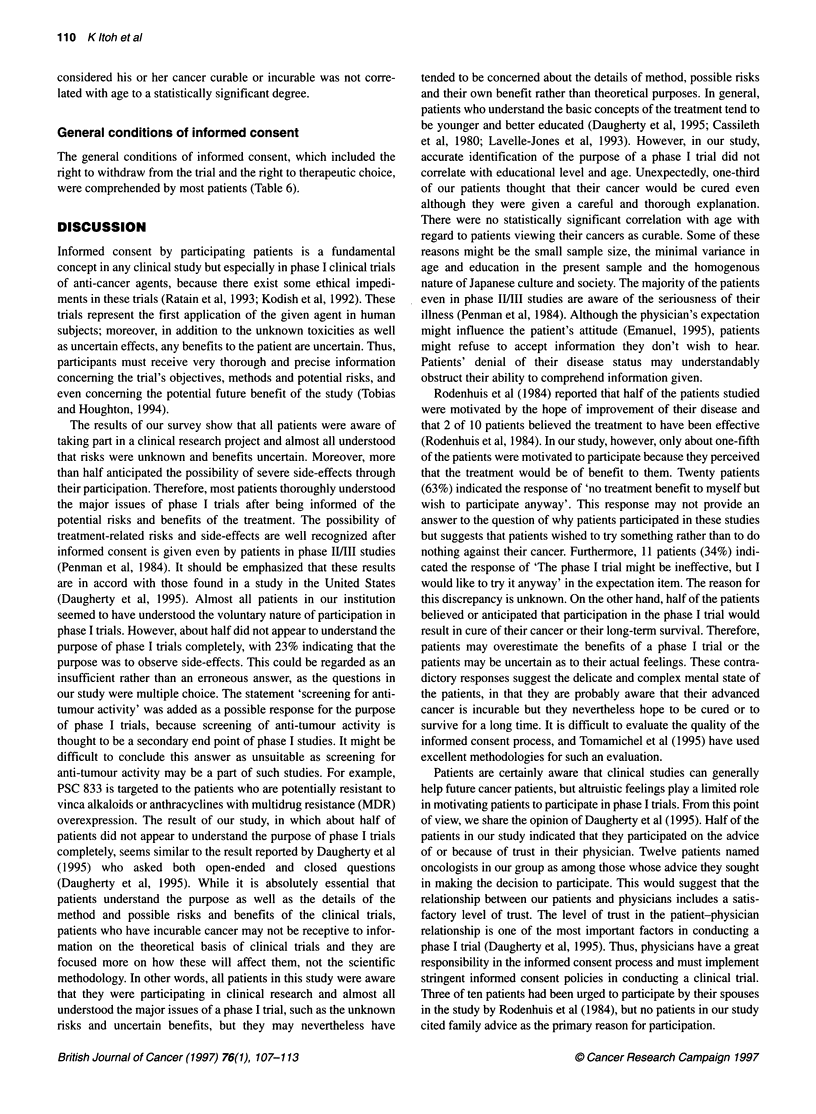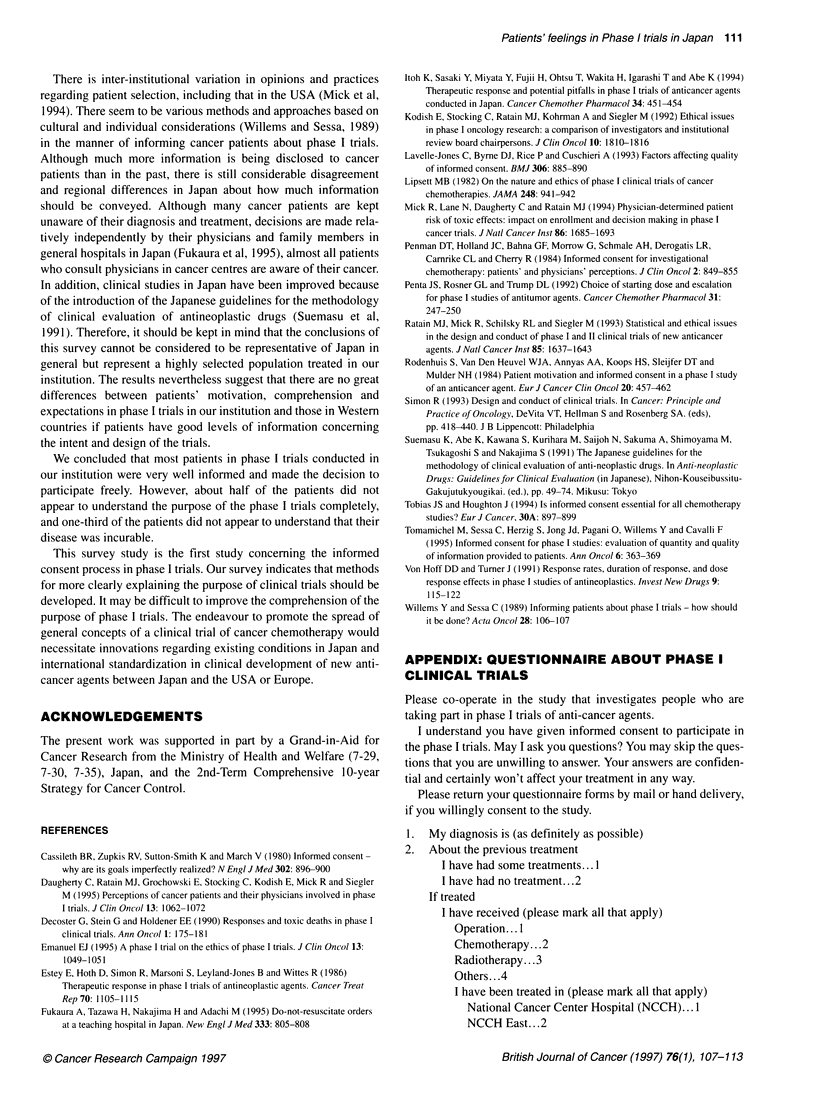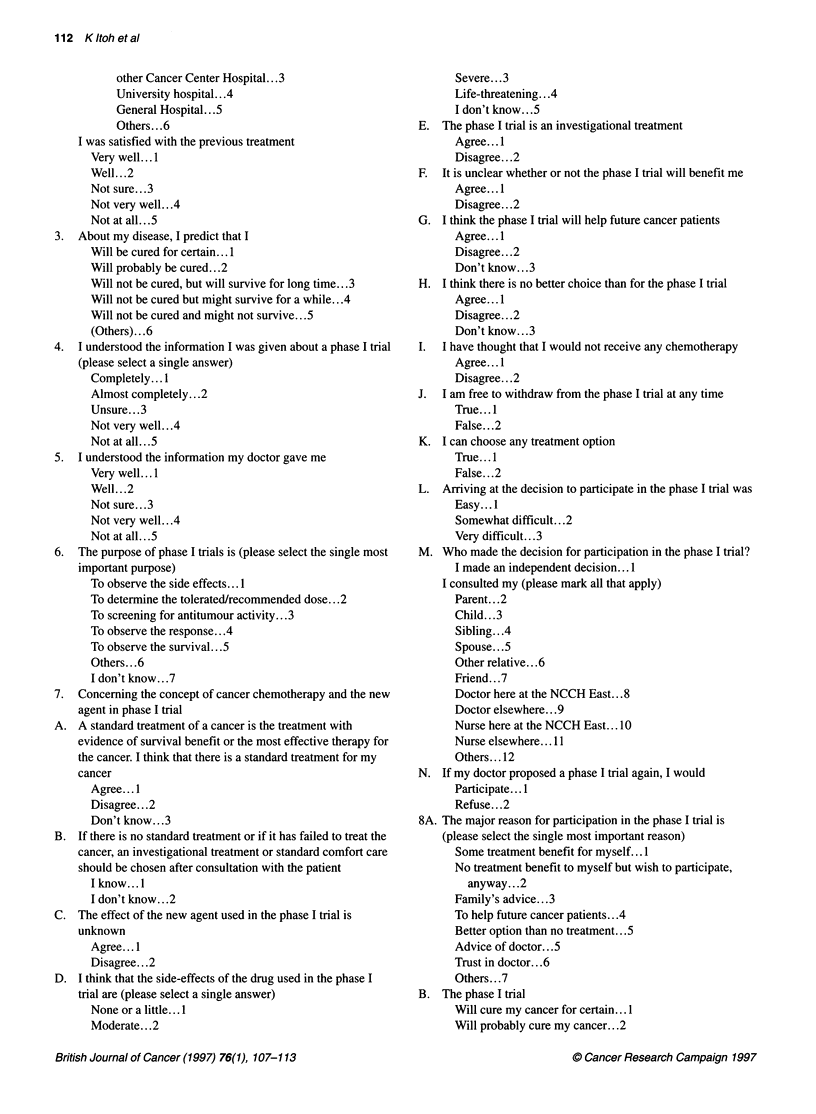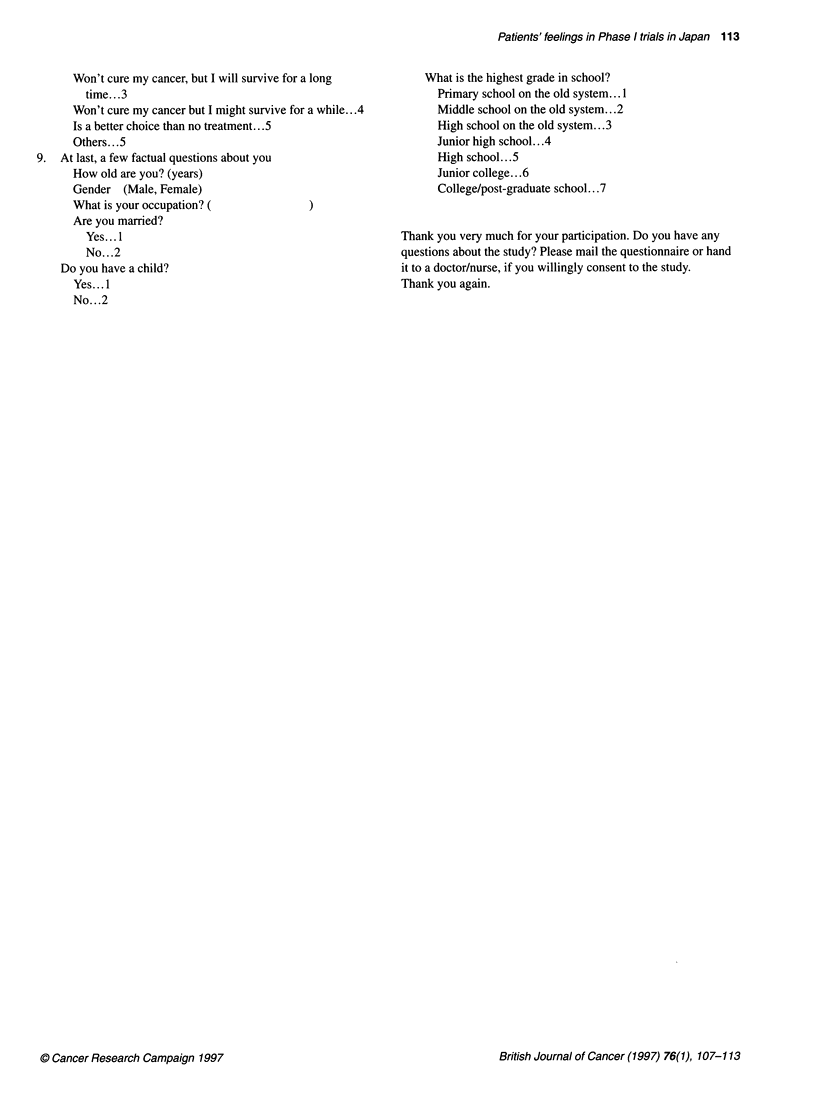# Patients in phase I trials of anti-cancer agents in Japan: motivation, comprehension and expectations.

**DOI:** 10.1038/bjc.1997.344

**Published:** 1997

**Authors:** K. Itoh, Y. Sasaki, H. Fujii, T. Ohtsu, H. Wakita, T. Igarashi, K. Abe

**Affiliations:** Department of Medicine, National Cancer Center Hospital East, Kashiwa, Chiba, Japan.

## Abstract

We attempted to characterize the motivation, comprehension and expectations of patients who had given informed consent to participate in phase I trials of anti-cancer agents at the National Cancer Center of Japan. Thirty-three patients were given a simple multiple-choice questionnaire and asked to return it at a later date. The completed survey was returned by 32 patients. The patients were surveyed before they had received any investigational phase I agents. Nineteen per cent of patients were motivated to participate in the phase I trials by the possibility of therapeutic benefit, 9% because participation seemed a better choice than no treatment and only 6% for altruistic reasons. Most patients comprehended the major features of a phase I trial, namely its investigational nature, the unknown effects of the agent investigated and the unclear benefit to the patients themselves. Fifty-nine per cent of the patients anticipated that they might suffer severe or life-threatening side-effects if they participated in the phase I trial, and 43% were able to indicate accurately the purpose of the phase I trial as a dose determination study. Although only a minority of the patients indicated that their motivation to participate was possible treatment benefit to themselves, when answering questions regarding expectations, more than half indicated that there might be personal benefits of varying degrees by participation.


British Journal of Cancer (1997) 76(1), 107–113
© 1997 Cancer Research Campaign

### Patients in phase I trials of anti-cancer agents in Japan: motivation, comprehension and expectations

**K Itoh, Y Sasaki, H Fujii, T Ohtsu, H Wakita, T Igarashi and K Abe**

Division of Oncology/Hematology, Department of Medicine, National Cancer Center Hospital East, Kashiwanoha 6-5-1, Kashiwa, Chiba 277, Japan

**Summary** We attempted to characterize the motivation, comprehension and expectations of patients who had given informed consent to participate in phase I trials of anti-cancer agents at the National Cancer Center of Japan. Thirty-three patients were given a simple multiple-choice questionnaire and asked to return it at a later date. The completed survey was returned by 32 patients. The patients were surveyed before they had received any investigational phase I agents. Nineteen per cent of patients were motivated to participate in the phase I trials by the possibility of therapeutic benefit, 9% because participation seemed a better choice than no treatment and only 6% for altruistic reasons. Most patients comprehended the major features of a phase I trial, namely its investigational nature, the unknown effects of the agent investigated and the unclear benefit to the patients themselves. Fifty-nine per cent of the patients anticipated that they might suffer severe or life-threatening side-effects if they participated in the phase I trial, and 43% were able to indicate accurately the purpose of the phase I trial as a dose determination study. Although only a minority of the patients indicated that their motivation to participate was possible treatment benefit to themselves, when answering questions regarding expectations, more than half indicated that there might be personal benefits of varying degrees by participation.

**Keywords:** questionnaire; phase I trials; patients' feelings

The primary goals of phase I clinical trials for anti-cancer agents are to determine toxicity, maximum tolerated dose and recommended dose for the phase II study, as well as pharmacological evaluation of the new drug (Simon, 1993). Preliminary evaluation of anti-tumour activity may also be included in phase I clinical trials.

A phase I clinical trial is the initial trial in human subjects of a new agent. Although healthy volunteers are appropriate candidates for phase I trials, this generalization does not apply to phase I trials of anti-cancer agents because of the side-effects and narrow therapeutic window. Anti-cancer agents in phase I trials should always be administered to patients with incurable cancer with the expectation of some therapeutic benefit. Accordingly, it is not surprising that toxicity and possible benefits of such agents are often unknown, even when there is promising preclinical data. In addition, in phase I trials, early cohorts of patients are treated at very low and sometimes ineffective dosages. The overall response rate in phase I trials is low (Estey et al, 1986; Decoster et al, 1990; Von Hoff and Turner, 1991; Penta et al, 1992; Itoh et al, 1994). Furthermore, physicians as investigators conducting phase I trials of anti-cancer agents must always be vigilant in safeguarding the patients' best interests when confronted with ethical issues (e.g. benefit vs toxicity), and, moreover, obtaining truly informed consent is difficult (Lipsett, 1982; Ratain et al, 1993; Emanuel, 1995).

Although almost all investigators and institutional review boards agree as to the importance of informed consent (Kodish et al, 1992) and the guiding concept in ethical clinical research is informed consent, which is meant to guarantee the voluntary nature of participation in clinical trials, there have been only a few reports from the viewpoint of the patients in phase I clinical trials of anti-cancer agents (Rodenhuis et al, 1984; Tomamichel et al, 1995; Daugherty et al, 1995). Furthermore, it is of interest to learn whether the patients' perception of these studies in Japan differs from that of patients in the United States and Europe. We administered a questionnaire to 33 consecutive patients who had given informed consent to participate in a phase I trial of anti-cancer agents at the National Cancer Center Hospital East in an attempt to characterize their motivation, comprehension and expectations.

*Received 31 May 1996*
*Revised 25 November 1996*
*Accepted 11 December 1996*

*Correspondence to:* K Itoh

### PATIENTS AND METHODS

The subjects were 33 patients with advanced or metastatic cancer who had given informed consent consecutively to participate in one of three phase I trials of anti-cancer agents [NB506 (Banyu Pharmaceutical), SZS-PSC833 in combination with doxorubicin (Sandoz Pharmaceuticals), JM216 (Bristol-Myers Squibb)] conducted at the National Cancer Center Hospital East between 23 June 1994 and 21 September 1995. Patients were asked if they would agree to participate in a survey by completing a questionnaire. Once verbal approval was obtained, a simple multiple-choice questionnaire was distributed, with assurance of anonymity, before any treatment with the agents under investigation was administered but after they received detailed information concerning the objectives of the trial. In addition to the information provided in the consent forms, patients were verbally informed firstly as to the incurable nature of their cancer, for which no standard treatment was available, secondly that a phase I clinical trial is the first trial in human subjects for research purposes on effectiveness of an anti-cancer agent and, finally, of the objectives, methods, potential risks and uncertain effects of the trial. In addition, general information about the clinical study was

Table 1 Demographics of the surveyed patients ($n = 32$)

| Characteristic | No. of patients |
|---|---|
| Gender | |
| Male | 15 |
| Female | 17 |
| Age (median 58 years) | |
| <49 | 9 |
| 50–59 | 10 |
| >60 | 13 |
| Occupation | |
| None/retired | 14 |
| White collar/professional | 12 |
| Not described | 6 |
| Education | |
| Did not attend high school | 5 |
| High school | 15 |
| Beyond high school | 9 |
| Unknown | 3 |
| Malignant tumour | |
| Gastrointestinal tract | 9 |
| Breast | 7 |
| Head and neck | 5 |
| Lung | 3 |
| Gynaecourinary | 3 |
| Others | 5 |
| Prior therapy | |
| None | 1 |
| Operation ± radiotherapy | 3 |
| Chemotherapy ± radiotherapy | 9 |
| Operation + chemotherapy ± radiotherapy | 19 |

provided. The no treatment option that was included in the written consent form was explained. The information regarding the phase I trial was standardized in the written consent forms and physicians used this information to reduce variance of the verbal presentation among the six staff physicians involved in the study. The questionnaires were returned by mail or hand delivered to each physician and were considered as confidential information. A single physician managed the entire process of the distribution and recovery of the survey forms.

All patients who were candidates for phase I trials had a histologically confirmed malignancy for which there was no effective chemotherapy or which had been demonstrated to be refractory to standard therapy. Other eligibility criteria in the three phase I trials were: patient must be older than 20 years; a good performance status (PS) [PS 0–2 by Eastern Cooperative Oncology Group (ECOG)]; no radiation therapy or chemotherapy within 4 weeks before entry into the trials; adequate organ function; no serious complications; and written informed consent.

The questionnaire study was approved by the Institutional Review Board and Ethical Committee at the National Cancer Center in Japan. Statistical analysis was performed using Fisher's exact probability test in Stat View.

### RESULTS

All 33 patients returned the questionnaires, but one patient failed to complete the survey, leaving 32 completed forms for analysis. The characteristics of the 32 patients are summarized in Table 1. Median age was 58 years (range 30–68 years), and 53% were women. Four patients were unable to participate in phase I trials because neutropenia was detected before registration. All patients except one had received some previous treatment.

Table 2 Motivations for participation in the phase I trial

| | n | (%) |
|---|---|---|
| The major reason for participation in the phase I trial is[a] | | |
| Some treatment benefit for myself | 6 | (19) |
| No treatment benefit to myself but wish to participate anyway | 20 | (63) |
| Family's advice | 0 | (0) |
| To help future cancer patients | 2 | (6) |
| Better option than no treatment | 3 | (9) |
| Advice of doctor | 7 | (22) |
| Trust in doctor | 9 | (28) |
| If my doctor proposed a phase I trial again, I would | | |
| Participate | 19 | (73) |
| Refuse | 1 | (4) |
| Don't know | 6 | (23) |
| I think the phase I trial will help future cancer patients | | |
| Agree | 28 | (90) |
| Disagree | 0 | (0) |
| Don't know | 3 | (10) |

[a]Although patients were instructed to select the single most important reason, some patients selected more than one reason.

Table 3 Patients' decision-making for participation in the phase I trial

| | n | (%) |
|---|---|---|
| Arriving at the decision to participate in the phase I trial was | | |
| Easy | 19 | (63) |
| Somewhat difficult | 7 | (23) |
| Very difficult | 4 | (13) |
| I made an independent decision | 2 | (7) |
| I consulted my (include any of the following) | | |
| Spouse | 24 | (80) |
| Child | 17 | (57) |
| Parent | 2 | (7) |
| Sibling | 4 | (13) |
| Other relative | 4 | (13) |
| Friend | 5 | (17) |
| Doctor | 13 | (43) |
| Nurse | 1 | (3) |

### Perception

With regard to patient's perception of the lack of or the failure of a standard treatment in treating the cancer, 25 patients (86%) understood the concept that an investigational treatment or standard comfort care should be chosen by the patient after consultation with the physician if there is no standard treatment or if the treatment has failed to treat the cancer.

### Motivation

As shown in Table 2, 20 patients (63%) indicated that they did not expect any benefit but wished to participate anyway. Furthermore, 11 of these 20 patients said that they would participate in another phase I trial if their doctor suggested it. Three patients (9%) indicated that a phase I trial was a better choice than no treatment at

© Cancer Research Campaign 1997

**Table 4** Patients' comprehension of the basic features of a phase I trial

| | *n* | (%) |
|---|---|---|
| I understood the information I was given about a phase I trial | | |
| Completely | 3 | (10) |
| Almost completely | 22 | (71) |
| Unsure | 4 | (13) |
| Not very well | 2 | (6) |
| Not at all | 0 | (0) |
| I understood the information my doctor gave me | | |
| Very well | 6 | (20) |
| Well | 22 | (73) |
| Not sure | 1 | (3) |
| Not very well | 1 | (3) |
| Not at all | 0 | (0) |
| The phase I trial is an investigational treatment | | |
| Agree | 31 | (100) |
| Disagree | 0 | (0) |
| The effect of the new agent used in the phase I trial is unknown | | |
| Agree | 30 | (97) |
| Disagree | 1 | (3) |
| It is unclear whether or not the phase I trial will benefit me | | |
| Agree | 29 | (94) |
| Disagree | 2 | (6) |
| I think that the side-effects of the drug used in the phase I trial are | | |
| None or a little | 0 | (0) |
| Moderate | 12 | (38) |
| Severe | 16 | (50) |
| Life-threatening | 3 | (9) |
| I don't know | 1 | (3) |
| The purpose of phase I trials is[a] | | |
| To observe the side-effects | 7 | (23) |
| To determine the tolerated/recommended dose | 13 | (43) |
| To screening for anti-tumour activity | 2 | (7) |
| To observe the response | 12 | (40) |
| To observe the survival | 0 | |

[a]Although patients were instructed to select the single most important reason, some patients selected more than one reason.

**Table 5** Patients' expectations for the phase I trial

| | *n* | (%) |
|---|---|---|
| I think there is no better choice than for the phase I trial | | |
| Agree | 13 | (42) |
| Disagree | 6 | (19) |
| Don't know | 12 | (39) |
| I have thought that I would not receive any chemotherapy | | |
| Agree | 15 | (50) |
| Disagree | 15 | (50) |
| The phase I trial (check as many as apply) | | |
| Will cure my cancer for certain | 6 | (19) |
| Will probably cure my cancer | 6 | (19) |
| Won't cure my cancer, but I will survive for a long time | 5 | (16) |
| Won't cure my cancer, but I might survive for a while | 3 | (9) |
| Might be ineffective, but I would like to try it anyway | 11 | (34) |
| Is a better choice than no treatment | 5 | (16) |
| I predict that I | | |
| Will be cured for certain | 5 | (16) |
| Will probably be cured | 6 | (19) |
| Will not be cured but will survive for a long time | 8 | (26) |
| Will not be cured but might survive for a while | 8 | (26) |
| Will not be cured and might not survive | 4 | (13) |

**Table 6** General issues of informed consent

| | *n* | (%) |
|---|---|---|
| I am free to withdraw from the phase I trial at any time | | |
| True | 30 | (97) |
| False | 1 | (3) |
| I can choose any treatment option | | |
| True | 23 | (88) |
| False | 3 | (12) |

all, and 16(50%) indicated that they decided to participate on the advice of or because of their trust in their physician. Furthermore, six patients (19%) were motivated in their decision to participate by the possibility of therapeutic benefit for themselves. Altruistic reasons were given by only two patients (6%) as the primary reason for participating, although most patients (90%) considered that the phase I trials would help future cancer patients.

Although arriving at the decision to participate in the phase I trial was easy for many patients (63%), only two patients made the decision independently without consulting with family members, friends or physicians (Table 3).

### Comprehension

The patients' comprehension of the basic features of the phase I trial is shown in Table 4. Most of the patients indicated that they understood all (10%) or almost all (71%) of the information provided about the trial in which they would participate, and 93% indicated that they could understand the explanation given by their doctors. Most patients comprehended the basic nature of a phase I trial of anti-cancer agents in that it is an investigation of a drug of unknown effect and of unclear benefit to the patients themselves. Nineteen patients (59%) anticipated that they might suffer severe (50%) or life-threatening (9%) side-effects if they participated in the phase I trial. All patients anticipated some side-effects. Almost half of the patients were able to indicate accurately that the purpose of the phase I trial in which they were scheduled to participate was a dose-determination study. However, 12 patients (40%) considered that the purpose of the phase I trial was to observe the response. The accurate identification of the purpose of the phase I trial was not correlated with the education level. Two of nine (22%) patients who had a college-level or higher education indicated the purpose of the phase I trial, as opposed to 11 of 20 (55%) educated to high school level or below. Similarly, the frequency of the correct response was not correlated with age (more than or less than 60 years) or with gender.

### Expectations

Thirteen patients (42%) agreed that there was no better choice than the phase I trial. To consider the option of no chemotherapy was not correlated with age (more than or less than 60 years) or gender. Eleven patients (34%) indicated that they wanted to participate even if the phase I trial turned out to be ineffective. About one-third of the patients (35%) considered that their cancer would be cured. Moreover, 12 expected to be cured in the phase I trial (Table 5). Six of 13 (46%) patients who were older than 60 years indicated that they considered their cancer curable, as opposed to 5 of 18 (28%) of those younger than 60 years. Whether a patient

© Cancer Research Campaign 1997

considered his or her cancer curable or incurable was not correlated with age to a statistically significant degree.

### General conditions of informed consent

The general conditions of informed consent, which included the right to withdraw from the trial and the right to therapeutic choice, were comprehended by most patients (Table 6).

### DISCUSSION

Informed consent by participating patients is a fundamental concept in any clinical study but especially in phase I clinical trials of anti-cancer agents, because there exist some ethical impediments in these trials (Ratain et al, 1993; Kodish et al, 1992). These trials represent the first application of the given agent in human subjects; moreover, in addition to the unknown toxicities as well as uncertain effects, any benefits to the patient are uncertain. Thus, participants must receive very thorough and precise information concerning the trial's objectives, methods and potential risks, and even concerning the potential future benefit of the study (Tobias and Houghton, 1994).

The results of our survey show that all patients were aware of taking part in a clinical research project and almost all understood that risks were unknown and benefits uncertain. Moreover, more than half anticipated the possibility of severe side-effects through their participation. Therefore, most patients thoroughly understood the major issues of phase I trials after being informed of the potential risks and benefits of the treatment. The possibility of treatment-related risks and side-effects are well recognized after informed consent is given even by patients in phase II/III studies (Penman et al, 1984). It should be emphasized that these results are in accord with those found in a study in the United States (Daugherty et al, 1995). Almost all patients in our institution seemed to have understood the voluntary nature of participation in phase I trials. However, about half did not appear to understand the purpose of phase I trials completely, with 23% indicating that the purpose was to observe side-effects. This could be regarded as an insufficient rather than an erroneous answer, as the questions in our study were multiple choice. The statement 'screening for anti-tumour activity' was added as a possible response for the purpose of phase I trials, because screening of anti-tumour activity is thought to be a secondary end point of phase I studies. It might be difficult to conclude this answer as unsuitable as screening for anti-tumour activity may be a part of such studies. For example, PSC 833 is targeted to the patients who are potentially resistant to vinca alkaloids or anthracyclines with multidrug resistance (MDR) overexpression. The result of our study, in which about half of patients did not appear to understand the purpose of phase I trials completely, seems similar to the result reported by Daugherty et al (1995) who asked both open-ended and closed questions (Daugherty et al, 1995). While it is absolutely essential that patients understand the purpose as well as the details of the method and possible risks and benefits of the clinical trials, patients who have incurable cancer may not be receptive to information on the theoretical basis of clinical trials and they are focused more on how these will affect them, not the scientific methodology. In other words, all patients in this study were aware that they were participating in clinical research and almost all understood the major issues of a phase I trial, such as the unknown risks and uncertain benefits, but they may nevertheless have tended to be concerned about the details of method, possible risks and their own benefit rather than theoretical purposes. In general, patients who understand the basic concepts of the treatment tend to be younger and better educated (Daugherty et al, 1995; Cassileth et al, 1980; Lavelle-Jones et al, 1993). However, in our study, accurate identification of the purpose of a phase I trial did not correlate with educational level and age. Unexpectedly, one-third of our patients thought that their cancer would be cured even although they were given a careful and thorough explanation. There were no statistically significant correlation with age with regard to patients viewing their cancers as curable. Some of these reasons might be the small sample size, the minimal variance in age and education in the present sample and the homogenous nature of Japanese culture and society. The majority of the patients even in phase II/III studies are aware of the seriousness of their illness (Penman et al, 1984). Although the physician's expectation might influence the patient's attitude (Emanuel, 1995), patients might refuse to accept information they don't wish to hear. Patients' denial of their disease status may understandably obstruct their ability to comprehend information given.

Rodenhuis et al (1984) reported that half of the patients studied were motivated by the hope of improvement of their disease and that 2 of 10 patients believed the treatment to have been effective (Rodenhuis et al, 1984). In our study, however, only about one-fifth of the patients were motivated to participate because they perceived that the treatment would be of benefit to them. Twenty patients (63%) indicated the response of 'no treatment benefit to myself but wish to participate anyway'. This response may not provide an answer to the question of why patients participated in these studies but suggests that patients wished to try something rather than to do nothing against their cancer. Furthermore, 11 patients (34%) indicated the response of 'The phase I trial might be ineffective, but I would like to try it anyway' in the expectation item. The reason for this discrepancy is unknown. On the other hand, half of the patients believed or anticipated that participation in the phase I trial would result in cure of their cancer or their long-term survival. Therefore, patients may overestimate the benefits of a phase I trial or the patients may be uncertain as to their actual feelings. These contradictory responses suggest the delicate and complex mental state of the patients, in that they are probably aware that their advanced cancer is incurable but they nevertheless hope to be cured or to survive for a long time. It is difficult to evaluate the quality of the informed consent process, and Tomamichel et al (1995) have used excellent methodologies for such an evaluation.

Patients are certainly aware that clinical studies can generally help future cancer patients, but altruistic feelings play a limited role in motivating patients to participate in phase I trials. From this point of view, we share the opinion of Daugherty et al (1995). Half of the patients in our study indicated that they participated on the advice of or because of trust in their physician. Twelve patients named oncologists in our group as among those whose advice they sought in making the decision to participate. This would suggest that the relationship between our patients and physicians includes a satisfactory level of trust. The level of trust in the patient–physician relationship is one of the most important factors in conducting a phase I trial (Daugherty et al, 1995). Thus, physicians have a great responsibility in the informed consent process and must implement stringent informed consent policies in conducting a clinical trial. Three of ten patients had been urged to participate by their spouses in the study by Rodenhuis et al (1984), but no patients in our study cited family advice as the primary reason for participation.

© Cancer Research Campaign 1997

There is inter-institutional variation in opinions and practices regarding patient selection, including that in the USA (Mick et al, 1994). There seem to be various methods and approaches based on cultural and individual considerations (Willems and Sessa, 1989) in the manner of informing cancer patients about phase I trials. Although much more information is being disclosed to cancer patients than in the past, there is still considerable disagreement and regional differences in Japan about how much information should be conveyed. Although many cancer patients are kept unaware of their diagnosis and treatment, decisions are made relatively independently by their physicians and family members in general hospitals in Japan (Fukaura et al, 1995), almost all patients who consult physicians in cancer centres are aware of their cancer. In addition, clinical studies in Japan have been improved because of the introduction of the Japanese guidelines for the methodology of clinical evaluation of antineoplastic drugs (Suemasu et al, 1991). Therefore, it should be kept in mind that the conclusions of this survey cannot be considered to be representative of Japan in general but represent a highly selected population treated in our institution. The results nevertheless suggest that there are no great differences between patients' motivation, comprehension and expectations in phase I trials in our institution and those in Western countries if patients have good levels of information concerning the intent and design of the trials.

We concluded that most patients in phase I trials conducted in our institution were very well informed and made the decision to participate freely. However, about half of the patients did not appear to understand the purpose of the phase I trials completely, and one-third of the patients did not appear to understand that their disease was incurable.

This survey study is the first study concerning the informed consent process in phase I trials. Our survey indicates that methods for more clearly explaining the purpose of clinical trials should be developed. It may be difficult to improve the comprehension of the purpose of phase I trials. The endeavour to promote the spread of general concepts of a clinical trial of cancer chemotherapy would necessitate innovations regarding existing conditions in Japan and international standardization in clinical development of new anti-cancer agents between Japan and the USA or Europe.

### ACKNOWLEDGEMENTS

The present work was supported in part by a Grand-in-Aid for Cancer Research from the Ministry of Health and Welfare (7-29, 7-30, 7-35), Japan, and the 2nd-Term Comprehensive 10-year Strategy for Cancer Control.

### REFERENCES

Cassileth BR, Zupkis RV, Sutton-Smith K and March V (1980) Informed consent – why are its goals imperfectly realized? *N Engl J Med* **302**: 896–900

Daugherty C, Ratain MJ, Grochowski E, Stocking C, Kodish E, Mick R and Siegler M (1995) Perceptions of cancer patients and their physicians involved in phase I trials. *J Clin Oncol* **13**: 1062–1072

Decoster G, Stein G and Holdener EE (1990) Responses and toxic deaths in phase I clinical trials. *Ann Oncol* **1**: 175–181

Emanuel EJ (1995) A phase I trial on the ethics of phase I trials. *J Clin Oncol* **13**: 1049–1051

Estey E, Hoth D, Simon R, Marsoni S, Leyland-Jones B and Wittes R (1986) Therapeutic response in phase I trials of antineoplastic agents. *Cancer Treat Rep* **70**: 1105–1115

Fukaura A, Tazawa H, Nakajima H and Adachi M (1995) Do-not-resuscitate orders at a teaching hospital in Japan. *New Engl J Med* **333**: 805–808

Itoh K, Sasaki Y, Miyata Y, Fujii H, Ohtsu T, Wakita H, Igarashi T and Abe K (1994) Therapeutic response and potential pitfalls in phase I trials of anticancer agents conducted in Japan. *Cancer Chemother Pharmacol* **34**: 451–454

Kodish E, Stocking C, Ratain MJ, Kohrman A and Siegler M (1992) Ethical issues in phase I oncology research: a comparison of investigators and institutional review board chairpersons. *J Clin Oncol* **10**: 1810–1816

Lavelle-Jones C, Byrne DJ, Rice P and Cuschieri A (1993) Factors affecting quality of informed consent. *BMJ* **306**: 885–890

Lipsett MB (1982) On the nature and ethics of phase I clinical trials of cancer chemotherapies. *JAMA* **248**: 941–942

Mick R, Lane N, Daugherty C and Ratain MJ (1994) Physician-determined patient risk of toxic effects: impact on enrollment and decision making in phase I cancer trials. *J Natl Cancer Inst* **86**: 1685–1693

Penman DT, Holland JC, Bahna GF, Morrow G, Schmale AH, Derogatis LR, Carnrike CL and Cherry R (1984) Informed consent for investigational chemotherapy: patients' and physicians' perceptions. *J Clin Oncol* **2**: 849–855

Penta JS, Rosner GL and Trump DL (1992) Choice of starting dose and escalation for phase I studies of antitumor agents. *Cancer Chemother Pharmacol* **31**: 247–250

Ratain MJ, Mick R, Schilsky RL and Siegler M (1993) Statistical and ethical issues in the design and conduct of phase I and II clinical trials of new anticancer agents. *J Natl Cancer Inst* **85**: 1637–1643

Rodenhuis S, Van Den Heuvel WJA, Annyas AA, Koops HS, Sleijfer DT and Mulder NH (1984) Patient motivation and informed consent in a phase I study of an anticancer agent. *Eur J Cancer Clin Oncol* **20**: 457–462

Simon R (1993) Design and conduct of clinical trials. In *Cancer: Principle and Practice of Oncology*, DeVita VT, Hellman S and Rosenberg SA. (eds), pp. 418–440. J B Lippencott: Philadelphia

Suemasu K, Abe K, Kawana S, Kurihara M, Saijoh N, Sakuma A, Shimoyama M, Tsukagoshi S and Nakajima S (1991) The Japanese guidelines for the methodology of clinical evaluation of anti-neoplastic drugs. In *Anti-neoplastic Drugs: Guidelines for Clinical Evaluation* (in Japanese), Nihon-Kouseibussitu-Gakujutukyougikai. (ed.), pp. 49–74. Mikusu: Tokyo

Tobias JS and Houghton J (1994) Is informed consent essential for all chemotherapy studies? *Eur J Cancer*, **30A**: 897–899

Tomamichel M, Sessa C, Herzig S, Jong Jd, Pagani O, Willems Y and Cavalli F (1995) Informed consent for phase I studies: evaluation of quantity and quality of information provided to patients. *Ann Oncol* **6**: 363–369

Von Hoff DD and Turner J (1991) Response rates, duration of response, and dose response effects in phase I studies of antineoplastics. *Invest New Drugs* **9**: 115–122

Willems Y and Sessa C (1989) Informing patients about phase I trials – how should it be done? *Acta Oncol* **28**: 106–107

### APPENDIX: QUESTIONNAIRE ABOUT PHASE I CLINICAL TRIALS

Please co-operate in the study that investigates people who are taking part in phase I trials of anti-cancer agents.

I understand you have given informed consent to participate in the phase I trials. May I ask you questions? You may skip the questions that you are unwilling to answer. Your answers are confidential and certainly won't affect your treatment in any way.

Please return your questionnaire forms by mail or hand delivery, if you willingly consent to the study.

1. My diagnosis is (as definitely as possible)
2. About the previous treatment

    I have had some treatments...1

    I have had no treatment...2

    If treated

    I have received (please mark all that apply)

    Operation...1

    Chemotherapy...2

    Radiotherapy...3

    Others...4

    I have been treated in (please mark all that apply)

    National Cancer Center Hospital (NCCH)...1

    NCCH East...2

other Cancer Center Hospital...3
University hospital...4
General Hospital...5
Others...6

I was satisfied with the previous treatment
Very well...1
Well...2
Not sure...3
Not very well...4
Not at all...5

3. About my disease, I predict that I
Will be cured for certain...1
Will probably be cured...2
Will not be cured, but will survive for long time...3
Will not be cured but might survive for a while...4
Will not be cured and might not survive...5
(Others)...6

4. I understood the information I was given about a phase I trial (please select a single answer)
Completely...1
Almost completely...2
Unsure...3
Not very well...4
Not at all...5

5. I understood the information my doctor gave me
Very well...1
Well...2
Not sure...3
Not very well...4
Not at all...5

6. The purpose of phase I trials is (please select the single most important purpose)
To observe the side effects...1
To determine the tolerated/recommended dose...2
To screening for antitumour activity...3
To observe the response...4
To observe the survival...5
Others...6
I don't know...7

7. Concerning the concept of cancer chemotherapy and the new agent in phase I trial

A. A standard treatment of a cancer is the treatment with evidence of survival benefit or the most effective therapy for the cancer. I think that there is a standard treatment for my cancer
Agree...1
Disagree...2
Don't know...3

B. If there is no standard treatment or if it has failed to treat the cancer, an investigational treatment or standard comfort care should be chosen after consultation with the patient
I know...1
I don't know...2

C. The effect of the new agent used in the phase I trial is unknown
Agree...1
Disagree...2

D. I think that the side-effects of the drug used in the phase I trial are (please select a single answer)
None or a little...1
Moderate...2
Severe...3
Life-threatening...4
I don't know...5

E. The phase I trial is an investigational treatment
Agree...1
Disagree...2

F. It is unclear whether or not the phase I trial will benefit me
Agree...1
Disagree...2

G. I think the phase I trial will help future cancer patients
Agree...1
Disagree...2
Don't know...3

H. I think there is no better choice than for the phase I trial
Agree...1
Disagree...2
Don't know...3

I. I have thought that I would not receive any chemotherapy
Agree...1
Disagree...2

J. I am free to withdraw from the phase I trial at any time
True...1
False...2

K. I can choose any treatment option
True...1
False...2

L. Arriving at the decision to participate in the phase I trial was
Easy...1
Somewhat difficult...2
Very difficult...3

M. Who made the decision for participation in the phase I trial?
I made an independent decision...1
I consulted my (please mark all that apply)
Parent...2
Child...3
Sibling...4
Spouse...5
Other relative...6
Friend...7
Doctor here at the NCCH East...8
Doctor elsewhere...9
Nurse here at the NCCH East...10
Nurse elsewhere...11
Others...12

N. If my doctor proposed a phase I trial again, I would
Participate...1
Refuse...2

8A. The major reason for participation in the phase I trial is (please select the single most important reason)
Some treatment benefit for myself...1
No treatment benefit to myself but wish to participate, anyway...2
Family's advice...3
To help future cancer patients...4
Better option than no treatment...5
Advice of doctor...5
Trust in doctor...6
Others...7

B. The phase I trial
Will cure my cancer for certain...1
Will probably cure my cancer...2

© Cancer Research Campaign 1997

Won't cure my cancer, but I will survive for a long time...3
Won't cure my cancer but I might survive for a while...4
Is a better choice than no treatment...5
Others...5

9. At last, a few factual questions about you
How old are you? (years)
Gender (Male, Female)
What is your occupation? ( )
Are you married?
Yes...1
No...2
Do you have a child?
Yes...1
No...2
What is the highest grade in school?
Primary school on the old system...1
Middle school on the old system...2
High school on the old system...3
Junior high school...4
High school...5
Junior college...6
College/post-graduate school...7

Thank you very much for your participation. Do you have any questions about the study? Please mail the questionnaire or hand it to a doctor/nurse, if you willingly consent to the study.
Thank you again.

© Cancer Research Campaign 1997